# Suicide and Laser Refractive Surgery

**DOI:** 10.18502/jovr.v15i3.7464

**Published:** 2020-07-29

**Authors:** Ali Salimi, Edsel Ing, Nicholas Nianiaris

**Affiliations:** ^1^Department of Ophthalmology, McGill University, Montreal, Canada; ^2^Department of Ophthalmology, University of Toronto, Toronto, Canada

**Keywords:** Depression, Laser Refractive Surgery, LASIK, Suicide

Dear Editor,

Laser refractive surgery (LRS) is one of the most frequently performed and successful operations in medicine with 96% postoperative patient satisfaction.^[1]^ The possible sequelae of LRS include dry eye syndrome, blurred vision, glare, and night vision disturbance that are usually transient, but sometimes persist.^[1]^ Psychiatric complications such as psychosis, depression, suicidal ideation, attempted suicide or completed suicide (PDS) following LRS are rare,^[2]^ but generate marked media attention.^[3,4]^ Given the tragedy of suicide after LRS, we reviewed the PubMed, Embase, PsycINFO, and Google Scholar databases from inception to October 2019 using keywords and MeSH terms “laser refractive surgery” and “suicide”.

We found the details of six patients, mainly young men, who completed suicide after LRS (Table 1).^[2,4,5,6,7]^ The patient-support website lasikcomplications.com^[8]^ lists approximately 34 patients with PDS following LRS. From 2007 to 2018, approximately 8,230,000 LASIK procedures were performed in the United States.^[9]^ Given that, the incidence rate of completed suicide and PDS in the US is estimated to be 7 per 100,000,000 individuals and 4 per 10,000,000 individuals undergoing LRS per annum, respectively. In the US, the age-adjusted suicide rate has increased by 33% over the last two decades, with 13.9 suicides per 100,000 individuals reported in 2018.^[10]^ The proportion of patients with either completed suicide or PDS after LRS is markedly lower than the proportion of suicide in the general population (*P* < 0.001).

A thorough informed consent before LRS may help to exclude inappropriate surgical candidates. Although it is impossible to list every possible outcome after LRS, and postoperative suicide is extremely rare, under a patient-centered standard of informed consent, the mandate to disclose the possibility of PDS after LRS merits consideration. In addition, impaired vision and chronic pain were two of the five most common adverse outcomes resulting in legal disputes over duties to disclose treatment risks in a 2012 study from Australia.^[11]^

**Table 1:** Patients with completed suicide after LRS


**Author, Year**	**Age**	**Sex**	**Post-op eye pain**	**Blurred vision**	**Procedure**	**Latency between LRS and suicide**	**Clinical factors**

Favaro, 2018^[7]^	54	M	Yes	No	PRK	20 years	
Reindl, 2018^[4]^	35	F	Yes	Yes	SMILE	8 weeks	
FDA, 2016	27	M	Yes	Yes	PRK enhancement	1 year	Veteran. Post-traumatic stress disorder and depression
van Setten, 2015^[2]^	33	M	No	Subjective	LASIK	8 weeks	Pre-existing psychologic instability. Saw psychiatrist numerous times.
LASIKComplications.com, 2011^[8]^	54	M	Yes	Yes	LASIK	1 year	
Puglionesi, 2007^[5]^	28	M	No	Yes	LASIK	6.5 years	Pre-operative dry eyes, mydriasis and depressive symptoms.

M, male; F, female; LASIK, laser-assisted in situ keratomileusis; LRS, laser refractive surgery; SMILE, small incision lenticule extraction; PRK, photorefractive keratectomy; FDA, U.S. Food & Drug Administration. MAUDE Adverse Event Report: LASIK 2016 https://www.accessdata.fda.gov/scripts/cdrh/cfdocs/cfmaude/detail.cfm?mdrfoi__id=5434049 Eye pain, postoperative dry eye pain.							

Dry eye syndrome was associated with suicidal ideation at an odds ratio of 1.24 and LRS can exacerbate dry eye. Psychologic and pharmacologic predispositions to post-LRS dissatisfaction include preoperative depression, the use of retinoic acid and antidepressants, antipsychotics or hypnotics with anticholinergic activity that may compound dry eye symptoms in patients with LRS.^[12,13]^ Patients suffering from refractory pain after LASIK can be referred to clinics specializing in dry eye syndrome, scleral contact lenses, or chronic pain. Emergency psychiatric resources in addition to the hospital emergency room include psychiatry and suicide prevention hotlines.

In conclusion, suicide following LRS is exceedingly rare. Suicide is a complex mental health issue with a myriad of contributing factors, and to ascribe blame to LRS is a single cause fallacy. Various publications have reported that: (i) patients with compensated pre-existing psychiatric disorders showed no increased incidence of PDS postoperatively, (ii) mental health-related quality of life has been shown not to decrease after LRS, and (iii) LRS can improve psychological well-being.^[14]^

##  Financial Support and Sponsorship

Nil.

##  Conflicts of Interest

There are no conflicts of interest.

## References

[1] Eydelman M, Hilmantel G, Tarver M, Hofmeister E, May J, Hammel K, et al. Symptoms and satisfaction of patients in the patient-reported outcomes with laser in situ keratomileusis (PROWL) studies. *JAMA Ophthalmol* 2017;135:13–22. 10.1001/jamaophthalmol.2016.4587 27893066

[2] van Setten G. Suicide after excimer laser refractive surgery: on the importance of matching expectations. *Open J Ophthalmol *2015;5:145–148.

[3] W5. Canadian Ophthalmological Society and Canadian Cornea, External Disease and Refactive Surgery Society statement to CTV News and W5. CTV News [Internet]. 2019 April 5 [cited 2019 Oct 14]. Available from: https://www.ctvnews.ca/w5/canadian-ophthalmological-society-and-canadian-cornea-external-disease-and-refactive-surgery-society-statement-to-ctv-news-and-w5-1.4368071.

[4] Reindl J. Detroit meteorologist Jessica Starr's death by suicide puts focus on Lasik safety. Detroit Free Press [Internet]. 2018 Dec 18 [cited 2019 Oct 13]. Available from: https://www.usatoday.com/story/news/nation/2018/12/18/meteorologist-jessica-starr-death-suicide-lasik-questions/2346863002/.

[5] Puglionesi L. Man commits suicide on Haverford State grounds. Delaware County Times. 2007 July 6.

[6] O'Shea S, Leffler B, Willis-Owen G. This week on 16:9 – May 28 – 20/20 hindsight. Global News; 2011 August 5.

[7] Favaro A. Painful side-effects from laser eye surgery linked to man's suicide: family. CTV News [Internet]. 2018 Nov 28 [cited 2019 Oct 13]. Available from: https://www.ctvnews.ca/health/painful-side-effects-from-laser-eye-surgery-linked-to-man-s-suicide-family-1.4196890.

[8] LASIKComplications.com. LASIK complications. Are you considering LASIK, ReLEx SMILE? [Internet]. 2019 [cited 2019 Oct 14]. Available from: https://lasikcomplications.com/.

[9] Statista. Number of LASIK surgeries in the United States from 1996 to 2019 (in 1,000). [Internet]. 2016 July 18 [cited 2019 Oct 15]. Available from: https://www.statista.com/statistics/271478/number-of-lasik-surgeries-in-the-us/.

[10] United Health Foundation. Sucide in the United States, 2018 summary, America's health rankings annual report. [Internet]. 2018 [cited 2019 Oct 14]. Available from: https://www.americashealthrankings.org/explore/annual/measure/Suicide/state/ALL.

[11] Bismark M, Gogos A, Clark R, Gruen R, Gawande A, Studdert D. Legal disputes over duties to disclose treatment risks to patients: a review of negligence claims and complaints in Australia. *PLoS Med* 2012;9:e1001283. 10.1371/journal.pmed.1001283 PMC3413715 22879818

[12] Cabral-Macias J, Garcia-De la Rosa G, Rodriguez-Matilde D, Vela-Barrera I, Ledesma-Gil J, Ramirez-Miranda A, et al. Pressure-induced stromal keratopathy after laser in situ keratomileusis: Acute and late-onset presentations. *J Cataract Refract Surg* 2018;44:1284–1290. 10.1016/j.jcrs.2018.06.053 30107965

[13] Wong J, Lan W, Ong LM, Tong L. Non-hormonal systemic medications and dry eye. *Ocul Surf* 2011;9:212–226. 10.1016/s1542-0124(11)70034-9 22023816

[14] Awwad S, Alvarez-Chedzoy N, Bowman R, Cavanagh H, McCulley J. Quality of life changes after myopic wavefront-guided laser in situ keratomileusis. *Eye Contact Lens* 2009;35:128–132. 10.1097/ICL.0b013e3181a142e5 19421019

